# A mixed methods systematic review of the effects of patient online self-diagnosing in the ‘smart-phone society’ on the healthcare professional-patient relationship and medical authority

**DOI:** 10.1186/s12911-020-01243-6

**Published:** 2020-10-06

**Authors:** Annabel Farnood, Bridget Johnston, Frances S. Mair

**Affiliations:** 1 School of Medicine, Dentistry & Nursing, College of Medical, Veterinary & Life Sciences University of Glasgow, Glasgow, Scotland; 2 NHS Greater Glasgow and Clyde, Glasgow, Scotland; 3 General Practice and Primary Care, Institute of Health and Wellbeing, College of Medical, Veterinary & Life Sciences University of Glasgow, Glasgow, Scotland

**Keywords:** Online self-diagnosis, online health information, Medical information, Internet, Information seeking, Self-diagnosis, Digital health, Professional-patient relationship

## Abstract

**Background:**

As technology continues to advance, the internet is becoming increasingly popular. Self-diagnosis and health information seeking online is growing more common and it will be important to understand the influence this may have on the patient-healthcare professional relationship.

**Methods:**

A mixed-method systematic review of quantitative, qualitative and mixed method studies concerning the public and healthcare professionals’ perceptions of online self-diagnosis and health information seeking and how this can impact the patient-healthcare professional relationship. We searched MEDLINE, EMBASE, CINAHL, ACM & SCOPUS between 2007 and 2018. Relevant data were extracted, and a thematic analysis was conducted and conceptualised using the Normalisation Process Theory framework.

**Results:**

Of 6107 records identified, 25 articles met the review eligibility criteria which included 16 qualitative, 8 quantitative and 1 mixed method study. The findings indicated that patients found the internet as a complementary information source alongside healthcare professionals. Health care professionals were perceived to be the most reliable and valued information source. People feel responsible for their own health and find the internet to be a source that provides information rapidly with accessibility at their convenience. Most healthcare professionals agreed on the importance of collaboration with patients and the need to develop a partnership and shared decision-making process but struggled to find time in the consultation to do so efficiently. Some healthcare professionals felt that the internet was advantageous for patients looking after their own health, while others felt it was due to a lack of trust in their expertise. Patients tended to present information to the healthcare professional to support the therapeutic relationship rather than to challenge it and to become more involved in the decision-making process of their healthcare.

**Conclusion:**

The results of this review suggests that patients value healthcare professionals as a source of medical advice more than the internet. While health professionals’ views were mixed our findings indicate that online health information seeking can potentially improve the patient-healthcare professional relationship as patients reported they usually conducted an online search to form a partnership with the healthcare professional as opposed to trying to prove them wrong.

## Background

Online health information seeking and self-diagnosis is a growing phenomenon internationally [1]. Due to technology advances, the internet is more accessible than ever, with usage increasing, currently 84% of the US population now use online services [2]. The rising use of smartphones [3] and rapidly increasing availability of health information on the internet has led to more people using the internet as their first healthcare resource, often before seeking professional advice [4]. Consequently, there is growing interest in the effect of these changes in behaviour on health outcomes as well as the potential impact on the healthcare professional (HCP) and patient relationship.

A US survey reported that by 2013, more than one-third of US adults were searching online for medical information for self-diagnosis [5]. While a 2015 UK-wide survey of General Practitioners (GPs) reported that three-quarters of GPs have noticed an increase in people self-diagnosing online and, 21% have experienced people presenting with the information they have found online [6]. The main concern reported by GPs in this survey was that online self-diagnoses would lead to increased appointment-making by the ‘worried well’. It has also been suggested that doctors may feel intimidated by online self-diagnosis [7]. Concerns have also been raised from within the nursing profession that some of the information being accessed by people may be of poor quality, and instead of being based on robust clinical evidence, merely represents the commercial interests of the website owners [8].

Previous research on the topic of online self-diagnosis and health information seeking tends to focus on the quality of health information online [9] and the characteristics of online health information seekers [10]. Other research has explored patient satisfaction with HCP communication and patient-HCP interaction [11]. This systematic review investigates whether patient online self-diagnosis and health information seeking is affecting the patient-HCP relationship, and the perceptions of patients and HCPs regarding online self-diagnosis and searching for health information online. The patient-HCP relationship has been known to influence health outcomes and can improve the patient experience in the healthcare system [12, 13], hence efforts to understand whether and how this relationship may be influenced by increased health information seeking online is an important evidence gap.

This paper aims to address the following three research questions:

1. What are the effects of patients seeking online health information on the healthcare professional-patient relationship and medical authority?
2. How do healthcare professionals perceive patients’ use of online health information?
3. How do public/patients perceive the use of online health information?

## Methods

This mixed method systematic review was developed in accordance with the Preferred Reporting Items for Systematic Reviews and Meta-Analyses (PRISMA) quality requirements [14]. The protocol is registered on PROSPERO [15], the International Prospective Register of Systematic Reviews (CRD42018084230).

### Search strategy

The systematic literature search was conducted using five databases: MEDLINE, EMBASE, CINAHL, ACM and SCOPUS. All searches were conducted using an ‘advanced search’ functionality, restricted to English language only and published between 2007 and April 2018 (Table 1). Although self-diagnosis has been happening for many years, the smartphone has made this phenomenon increase due to the rapid and accessible health information that is available to consumers online [16]. Therefore, this date range was chosen to bring the results in line with the launch of the first Apple smartphone, ‘Apple iPhone’ in 2007 [15]. The search strategies were conducted using database specific controlled vocabularies and free text terms. The search terms, among others, included ‘information seeking behaviour’, ‘online self-diagnosis’, ‘internet’, ‘professional-patient relations’ and ‘mobile app’. There is not one universal term to describe internet use for health information. Therefore, it was important to search for both self-diagnosis and information seeking behaviour terms, as they can both indicate different types of internet use. Information seeking may be someone that is already diagnosed but wants to know more information about a specific condition. Self-diagnosis is seeking either the initial diagnosis, or a different diagnosis. These different search terms can show a variety of information platforms being used. ‘Endnote X7’ was used to remove duplicate citations before screening [17]. The full MEDLINE search strategy can be found in Appendix 1.

**Table 1:** Inclusion and exclusion criteria

**Study type**	• Publication date from 2007 - present • English language only • Studies that report primary data (qualitative and quantitative studies), Studies can use any form of qualitative or quantitative methods.	Interest was in papers ranging from the years of 2007–2018 as the first Apple iPhone was created in 2007. As this is a mixed method systematic review, the inclusion of studies that report primary data and use any form of qualitative or quantitative methods were considered appropriate for eligibility. This is to offer a broader scope in answering the research questions, and a better representation of the range of research that has already been undertaken. Study types that were grey literature/ not published in a peer review journal, dissertations/thesis, secondary data analysis, published abstracts, conference proceedings, commentary articles written to propose opinions and letters, or editorials were excluded from the review.
**Participants**	• Any individual (adult) over the age of 18. This includes patients, the public and health care professionals.	This study will only be reviewing adults aged 18 and over in order to maintain a generational research focus.
**Topic**	• Any physical health conditions. • Must be in relation to online self-diagnosing and health information seeking on the internet. • Can include any level of the diagnosis process – diagnosis, processing and treatment options. Can include the perceptions of the public and healthcare professionals on the topic. • Patient’s use of online forums to communicate health information with other patients.	There is currently a variety of health conditions being searched for on the internet, so this review aims to explore a range of different medical searches instead of specific conditions. Online forums are a commonly used medical resource, therefore were included for eligibility. Mental health was not eligible as this is a broad area and the focus was only on physical health conditions. Cancer and maternal health were excluded as these are both large specialty areas, therefore we focused on all other physical health conditions.
**Setting**	• Any ‘normal’ primary care setting (community, primary care clinics, home, online, education facilities).	Since online self-diagnosing can take place in any setting that has internet access or service areas, all normal type settings are deemed appropriate. The clinical setting was only focused in primary care and otherwise any setting outside the clinical area.

### Data screening / study selection process

Data screening was performed using a systematic review software named ‘DistillerSR’ [18]. Title and abstracts were screened by one researcher (AF). All full papers were screened independently by two reviewers (AF, BJ or FM).

### Data extraction

A standardised data extraction form adapted from Johnston et al. [19] was used to collect study characteristics for papers that met the eligibility criteria (see Additional files). If there was any uncertainty over the content and applicability of the data for the review, this was resolved through discussion within the team. The data extraction table is listed as Tables 6–8 in Additional files 1, 2, 3.

### Quality assessment of included studies

As this is a mixed method systematic review, a quality appraisal tool was required that could assess a diverse range of articles in a systematic way. The mixed methods appraisal tool (MMAT) [20] was chosen because it is designed specifically for mixed method studies and appraises qualitative, quantitative, mixed methods, and other types of empirical studies [21], which fits the criteria for this review. The tool is split into two sections: screening questions and the explanation phase. The mixed method appraisal tool discourages the use of a scoring system and instead offers a detailed presentation of the ratings to provide a better explanation of the quality of the included studies [21]. A spreadsheet template was used on Microsoft Excel with a ‘yes’ or ‘no’ answer system in order to gain a score percentage, followed by an explanation column to justify the quality assessment score.

Two reviewers (AF, BJ) independently assessed the quality of the eligible studies for reliability purposes. Discussions were engaged over any discrepancies, with a record kept of how the decisions were reached. All articles that met the study inclusion criteria were kept even if they were found to be methodologically weak based on the quality assessment, as they still have the potential to provide new and valuable insights in a field where the literature is relatively sparse.

### Data analysis/synthesis

The findings of qualitative and quantitative studies were tabulated separately.

The included studies were read, and a thematic analysis was undertaken to establish a list of themes and sub-themes [22]. Coding clinics were held to refine the themes identified. Each item of extracted data was coded independently through thematic analysis by researcher AF, and reviewed by two researchers (BJ, FM). Four themes were identified and were then mapped onto the constructs of the Normalisation Process Theory (NPT) [23] to aid conceptualisation of the data (Tables 2 and 3). Any data that fell outside the framework was noted to ensure there was no “shoe-horning” of themes into the framework. NPT is a useful framework to explain and understand how people integrate new interventions into their everyday routines [24]. It has four constructs: coherence; cognitive participation; collective action; and reflexive monitoring and has been successfully used in other systematic reviews [25, 26] (Table 2).

**Table 2:** Normalization Process Theory Core Constructs

Coherence (CO) (Sense-making work)	Cognitive Participation (CP) (Relationship work)	Collective Action (CA) (Enacting work)	Reflexive Monitoring (RM) (Appraisal work)
The sense-making work that people do individually and collectively when they are faced with online self-diagnosis and seeking online health information	The relational work that people do individually and collectively to build and sustain online health information seeking	The operational work that people do by investing effort and time to engage in online self-diagnosis and seeking online health information and to use this information in consultations	The appraisal work that people do when online self-diagnosing or seeking online health information that affects them and others around them
Differentiation:	Initiation	Interactional workability	Systemization
*How a set of practices are different from each other*	*Key participants driving a set of practices forward*	*Interactional work people do with each other in consultations and other everyday settings*	*Collecting information to determine how effective and useful it is*
Communal specification:	Enrolment	Relational integration	Communal appraisal
*A shared understanding of aims and benefits of a set of practices*	*Strategies used to engage in tasks and help secure implementation*	*Communicating reliable knowledge about tasks to build accountability and maintain confidence*	*Working together to determine and evaluate the worth of a set of practices*
Individual specification:	Legitimation	Skill set workability	Individual appraisal
*An understanding of the responsibilities around a task and practices*	*The belief that the set of practices is correct and if it is right to be involved*	*Task allocation and performances*	*Working as individuals to appraise the effects on themselves*
Internalization:	Activation	Contextual Integration	Reconfiguration
*Understanding the benefits and values of a set of practices*	*Defining actions, behaviours and procedures needed to sustain a practice and stay involved*	*Managing a set of practices through the allocation of different kinds of resources*	*Redefining procedures or modifying practices.*

**Table 3:** Normalization Process Theory Coding frame for the effects of online self-diagnosis on the patient-healthcare professional relationship

Coherence (Sense-Making Work)	Cognitive Participation (Relationship Work)	Collective Action (Enacting Work)	Reflexive Monitoring (Appraisal Work)
Differentiation	**Initiation**	**Interactional workability**	**Systemization**
Understanding the differences between peoples’ use of the internet for online self-diagnosis with the healthcare professional’s diagnosis.	HCPs communicating and recommending online health websites to people.	Bringing online health information to consultations and the effect on the consultation and communication between the patient and HCP.	Determining the benefits and risks of online self-diagnosis.
Communal specification	**Enrolment**	**Relational integration**	**Communal appraisal**
Using online health forums and communities to gain information and self-diagnose.	HCPs reactions and behaviours towards internet-informed patients.	The influence (e.g. on confidence) of bringing online information to the relationship between the HCP and internet-informed patients.	Sharing online health information with HCPs and how HCPs react to this.
Individual specification	**Legitimation**	**Skillset workability**	**Individual appraisal**
People achieving an understanding of health information gained through the internet.	HCPs perspectives of online self-diagnosis and if they believe this is beneficial or the right thing for people to do.	The effect of using online information on roles and responsibilities of members of the public or HCPs.	Judging the quality of online information; to what extent do the public or HCPs think the information on the internet is reliable and accurate?
Internalization	**Activation**	**Contextual integration**	**Reconfiguration**
Peoples understanding and perceptions of using the internet to self-diagnose and knowing if this is their preference or if they value the role of the HCP consultations instead.	Communicating effectively with internet-informed people and adapting behaviour towards them.	Integrating online self-diagnosis into social circumstances.	Understanding how online self-diagnosis affects the patient-HCP relationship and altering behaviour and reactions to ensure it is a positive change.

## Results

### Data screening / study selection process

Database searches retrieved 7026 papers in total which reduced to 6109 after deduplication. We had three phases of screening - title, abstract and full text. Each included a list of questions to pass each phase. 6109 titles were screened and 708 passed to abstract screening. Papers were removed in title screening if they were not relevant to the subject area, not in a peer reviewed journal and not involving humans. 708 abstracts were screened and 289 were assessed for full text screening. Abstracts were excluded for the same reasons in title screening, but also assessed for the correct setting, if they answered the research questions and if they were about physical health. The final number of papers deemed eligible to be included from the database search, was 23.

**Fig. 1:**
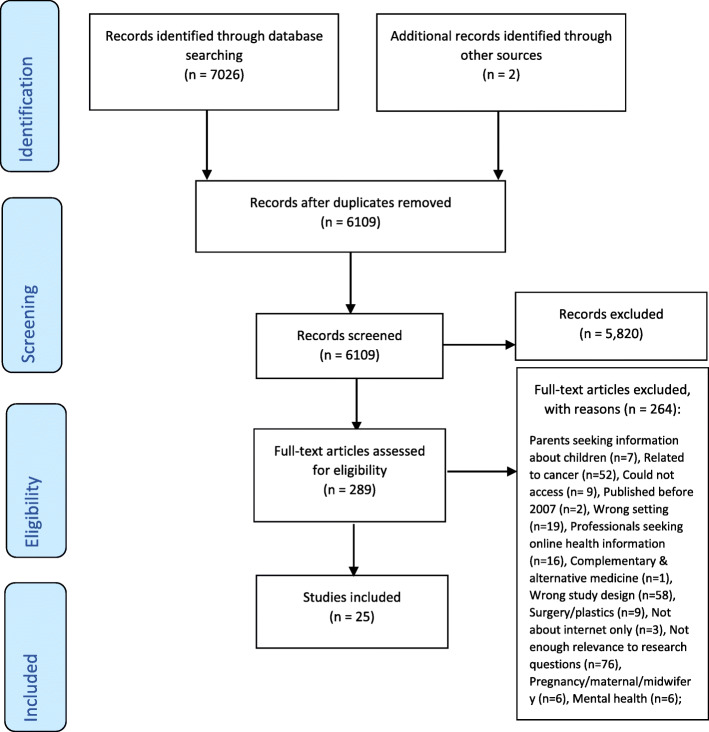
PRISMA flow diagram

Backward and forward chaining was implemented to ensure that no key articles were missed. During this process, another two papers were discovered to meet the eligibility criteria for review, making the final number of included papers in this mixed methods systematic review, 25 (see Fig. 1).

### Study characteristics

Of the 25 eligible papers, there were 16 qualitative, 8 quantitative and 1 mixed method study. (see Tables 6–8 in Additional files 1, 2, 3). Five studies had taken place in the United Kingdom [27–31], six from the United States [32–37], two from Canada [38, 39], one from Austria [40], four from Israel [41–44], two from Australia [45, 46], three from Switzerland [47–49], one from Portugal [50] and one from China [51]. The sample sizes ranged from 11 to 975 with participants including patients either posting online or attending primary care clinics, carers, physicians such as GP’s, nurses and various other HCP’s. Participants were from a variety of ages, genders, socio-economic groups and ethnicities. Although studies more frequently included middle-aged females and those of ‘white’ ethnicity. Full detail of study characteristics is provided in the data extraction tables (Tables 6–8 in Additional files 1, 2, 3) and further details of participant characteristics are provided (Table 9 in Additional file 4). Fewer studies explored the HCP’s perspectives compared to the patient’s perspectives.

### Quality assessment

The quality of reporting in the included studies varied and was measured using the mixed methods approach tool (MMAT). All 25 studies presented clear research questions and collected data to address the questions. Most of the qualitative studies used appropriate data collection methods to answer the research questions, reported the findings adequately derived from the data and provided coherence between qualitative data sources, collection, analysis and interpretation. Most quantitative studies used appropriate statistical analysis to answer the research questions and used appropriate measurements. Almost all the quantitative studies had pre-tested and piloted surveys before use. Fewer studies had samples that accurately represented the target population. Overall, the studies were of moderate quality. See the additional files for full quality assessment table.

### Data analysis/synthesis

Four major themes and several subthemes were identified from the synthesis of the literature. The four main themes are: 1) patient perspectives on using the internet to seek health information; 2) healthcare professionals’ perspectives on and reactions to internet-informed patients; 3) sharing online health information with healthcare professionals; 4) impact of online medical searches and diagnosis on patient-healthcare professional relationships (Table 4). Participant quotes are provided in the text to corroborate the data in each theme and are summarised in Table 5.

**Table 4:** Themes and sub-themes

**Theme 1: Patient perspectives on using the internet to seek health information**	
Subtheme 1: Reasons for using the internet	Why patients/public use the internet for healthcare advice.
Subtheme 2: Reasons against using the internet	Why patients/public are against using the internet for healthcare advice.
Subtheme 3: The prepared patient	Why patients/public felt the importance of being prepared for consultations and more informed of their health.
**Theme 2: Healthcare professionals’ perspectives on and reactions to internet-informed patients**	
Subtheme 1: HCP’s perceptions for and against people using the internet for online health information	HCP’s reasons for and against patient/public use of the internet for health information.
Subtheme 2: HCP’s reactions and behaviours to internet-informed patients	The importance of reactions and behaviours from HCP’s when faced with internet-informed patients.
**Theme 3: Sharing online health information with healthcare professionals**	
Subtheme 1: Communication	Enabling better communication within the consultation.
Subtheme 2: Bringing online health information to the consultation	The decision of whether patients/public would disclose or not disclose their online health information research to their HCP’s.
**Theme 4: Impact of online medical searches and diagnosis on patient-healthcare professional relationships**	
Subtheme 1: Trust	Patient/public’s trust in the internet and HCP’s.
Subtheme 2: Role changing	Change in the HCP-patient roles.
Subtheme 3: The patient-HCP relationship	How has online self-diagnosis affecting the patient-HCP relationship.

**Table 5:** Participant quotes supporting themes

Themes	Participant quotes
People’s perspectives of online self-diagnosis and online health information seeking Coherence (CO)	**Reasons for using the internet** • “I use the Internet at home and in the office, and it is very easy, easy and most of all rapid. You lose very little time.. . And when you find what you need, then you can come back later and in a little moment I can see all the new things. So, why should I not use it?” (Caiata-Zufferey et al., 2010 [49]) **Reasons against using the internet** • “There is so much information. For example, if I wanted information on healthy diet and how to lose weight, when you search, heaps and heaps of information comes up. So it’s really difficult to decide which to use, let alone whether it’s actually suitable for me or not, or even whether it’s trustworthy.” (Chu et al., 2017 [51]). **The prepared patient** • “… to go in feeling like you at least know maybe what to expect … and you know what questions to ask. Because sometimes going to the doctor is intimidating and then they … use the medical talk and you’re like, ‘I don’t really know what that means,’ so at least if you’ve read a little bit, you feel more prepared and can say, ‘Well, what about this?’” (Rupert et al., 2014 [33]).
Healthcare professionals’ perspectives of people online self-diagnosis and online health information seeking Cognitive Participation (CP)	**HCP’s perceptions for and against people using the internet for online health information** • “I think it is a good thing for patients to have access to medical information. … But this only applies to high-quality information. Because it makes people proactive. For instance, it makes people aware of insidious health problems that are often discovered too late.” (Caiata-Zufferey & Schulz, 2012 [48]). • “For me that was the irritation, that the patient had far more trust in the computer and what they found on the web than in what I was trying to explain.” (Ahluwalia et al., 2010 [28]). **HCP’s reactions and behaviours to internet-informed patients** • “I’ve … decided that right upfront if somebody has clearly done way more reading into an area that I’d ever done I just say: ‘Wow, you know more about that than I do’ … It’s really important not to feel threatened by that information because … if you [did] … that will affect your relationship” (Townsend et al., 2015 [38]).
Sharing online health information with healthcare professionals Cognitive Participation & Collective Action (CP & CA)	**Communication** • “a huge difference … finding information, and what it means, before you go to the doctor so you can have an intelligent conversation … [and] ask them the right questions” (Townsend et al., 2015 [38]). **Bringing online health information to the consultation** • “I kind of watch the way you say it because you don’t want to offend [doctors]. I would just kind of say ‘I didn’t know whether it could be this’ … and introduce it like that.” (Rupert et al., 2014 [33]). • “I think they [HCPs] probably take you a bit more seriously when you know your stuff, because they can’t fool you around, because they know that you have the answers” (Benetoli et al., 2018 [45]).
Impact of peoples use of the internet for self-diagnosis and health information seeking on their relationship with healthcare professionals Reflexive Monitoring (RM)	**Trust** • I wouldn’t trust a computer that much ... any specific information like ‘do this’ or ‘don’t do that’, because – even though it may be useful, I’d much rather deal with a human being, a doctor (Stevenson et al., 2007 [31]). • “If you spend that last 5 min … showing them [patients] … “This is a website that you can read too. It’s got enough information but not too much and it won’t overwhelm you. This is endorsed by the Canadian Arthritis Society.” It kind of builds a level of trust and … adds a component of enrichment to the appointment … they read about it and I think they just feel a lot more like, empowered and cared for … equipped.” (Townsend et al., 2015 [38]). **Role changing** • “That’s what I’ve been experiencing by now for the last 20 years; my professional authority isn’t as sacred as it used to be. I can’t say anymore that’s it, that’s what I see, this is what we know and the patients are trusting and believe that we know best. It’s no longer like this.” (Sommerhalder et al., 2009 [47]). **The patient-HCP relationship** • “It’s just helped me have … more of a conversation with my doctor rather than just being, you know, have a one-sided, just listening. I feel like I can be more active in that interaction.” (Rupert et al., 2014 [33]) • “You just have to be really open to the fact that they’re [patients] going to tell you things you didn’t know and that’s great. “Oh I hadn’t seen that before. That might be useful for me with other clients”. So I definitely feel it’s more of a partnership …” . (Townsend et al., 2015 [38]).

### Patient perspectives on using the internet to seek health information

People’s opinions of using the internet for self-diagnosis differ, leading to diverse views. Twelve qualitative studies [30, 31, 34, 37–39, 45–47, 49–51] and one mixed methods study [36], reported on this theme. Essentially, there were three sub-themes relating to: 1) why people used the internet to seek health information; 2) concerns about using the internet; 3) and a desire to be a “well informed” patient. These relate mostly to the NPT theoretical constructs of coherence (sense making) and reflexive monitoring (appraisal). However, some of the issues raised related to Collective Action (Enacting work) when considering the effort involved in searching for information online.

Essentially, the internet was thought to be informative, but there was evidence that people had concerns about the quality of the information available on the internet, with the belief it could be contradictory at times and should be seen as provisional [38]. Contradictory information could result in additional questions arising about health and trigger a seemingly endless cycle of information seeking [38, 39].

### Reasons for using the internet

Patients find the internet useful for finding out more information about their health conditions or the medications prescribed by their HCP [46]. Self-diagnosing symptoms while remaining anonymous such as by posting in online health forums was popular [37, 50] for a number of reasons. For example, people in countries where patients pay for their healthcare reported online self-diagnosis to be money saving and time efficient; they could access health information with ease for free as opposed to waiting for a healthcare appointment and then having to pay a fee [37].

Patients reported that the internet was often the first source they accessed for health information [37]. Patients found the internet convenient and that it allowed them to become more self-aware and to share their experiences within online health forums [33]. It allowed patients and the public to expand their knowledge and gain a deeper understanding of health information without involving their HCP. It was also seen as beneficial to revisit the information as many times as required for free [33]. The internet was generally seen as a tool for the treatment of non-serious medical issues or for self-diagnosis [40].

Accessibility and speed were key identified benefits of online self-diagnosing. The internet allows 24-h access, whereas obtaining an appointment with a HCP can be difficult [30, 31, 34].

*“The Internet is really easy to use, you can use it anytime. Unlike doctors or health clinics, I can’t call them and ask them at work, and after work, they are all closed. But with the Internet, you can search the information during work, and even after work, you can use your mobile phone to go on the Internet to search. I think this is really convenient and because it’s the Internet, it offers you more sources and opinions.”* [51]*.*

### Reasons against using the internet

There were reported concerns about the credibility, limitation and trustworthiness of online information [39, 51]. Difficulties included information overload and complex or contradictory information [47]. Searching for health information online demands time, energy, and physical effort, especially for those not as familiar with technology [38, 39, 51]. One qualitative study reported that many patients (31%) believed that advice taken from the internet was not personalised to their clinical situation or based on their past medical history, preventing accurate self-diagnosis [51]. The overwhelming amount of information online can also result in the masking of credible sources [50]. This impacts patients’ ability to depend on information and causes the public to find the internet less reliable than other sources of information such as HCPs [50]. However, most patients viewed their research as a complementary information source to be used alongside treatment from their HCP [30, 36, 47, 49, 50]. HCP’s were thus viewed as expert guides who could aid navigation through the otherwise overwhelming quantities of health-related internet information [38]. The internet was seen to work well as a means for self-diagnosis or to find information to help ease patients’ minds while awaiting doctors’ appointments [30].

*“I wouldn’t trust a computer that much … any specific information like ‘do this’ or ‘don’t do that’, because – even though it may be useful, I’d much rather deal with a human being, a doctor.”* [31]*.*

### The prepared patient

Some people saw online self-diagnosis as a method to increase their knowledge, making them better prepared and equipped for health consultations. Having a better understanding of symptoms and conditions was thought to help them to engage more effectively with HCP’s. It was also seen as a way to help them to make the most of the short time they have in consultations so they know what questions to ask [30, 38].

“*… to go in feeling like you at least know maybe what to expect … and you know what questions to ask. Because sometimes going to the doctor is intimidating and then they … use the medical talk and you’re like, ‘I don’t really know what that means,’ so at least if you’ve read a little bit, you feel more prepared and can say, ‘Well, what about this?”’* [33]*.*

It was also reported that some health knowledge acquired online, was beyond the expertise of General Practitioners (GPs), causing patients to feel the need to perform research themselves to improve their self-care [31]. Patients appreciated HCP’s evaluating their internet-derived health information carefully, as it helped them achieve clarity and certainty [47]. Nevertheless, Benetoli et al. [45] reported that their respondents felt that most HCP’s did not appreciate online health seeking behaviours.

### Healthcare professionals’ perspectives on and reactions to internet-informed patients

Patient self-diagnosis and the use of the internet for health information can also impact on a HCP’s role. Four qualitative [28, 38, 47, 48] and two quantitative [42–44], studies reported on these HCP’s perspectives and their behaviours and reactions towards internet-informed patients. There were two major themes in relation to this: 1) HCP perceptions; and HCP reactions and behaviours when dealing with internet-informed patients. These issues related to the NPT constructs of cognitive participations (relationship work); collective action (enacting work) and reflexive monitoring (appraisal work).

### HCP’s perceptions for and against people using the internet for online health information

Many HCP’s perceive online health information to be useful and beneficial in terms of the fact that it allows patients and the public access to a wealth of knowledge on health-related issues [28, 43, 47]. Two studies found that most nurses had positive attitudes (72.7%), to internet informed patients and that nurses previously exposed to consulting with internet-informed patients adopted more positive attitudes than those who had not been exposed [44]. Academic nurses and younger nurses reacted more comfortably to internet-informed patients compared to registered nurses and practical nurses. There was an overall positive reaction in nurses’ responses to internet-informed patients [42, 44]. Such reactions were more commonly found in those with academic degrees and higher self-epistemic authority and confidence [42, 44]. Many HCP’s reported that they could discuss information on a more medically grounded level with internet users than with non-internet users [47]. Some HCP’s felt that patients have a right to stay well-informed and that they are more satisfied this way. They also believed patients should take responsibility for their own health and be able to make decisions, provided that they can base these decisions on high-quality sources of information [48].

*“I think it is a good thing for patients to have access to medical information. … But this only applies to high-quality information. Because it makes people proactive. For instance, it makes people aware of insidious health problems that are often discovered too late.”* [48]*.*

However, some found online health information to be problematic, especially when patients/public interpretation of online health information was misleading or incorrect. Some physicians interpreted online health information seeking as suggesting a lack of trust in their medical expertise [28, 47]. There is also a known fear of losing control of the consultation with internet-informed patients and the feeling of being perceived as incompetent [28].

Internet-informed patients were also considered by some as potentially preventing a physician from being as effective as they could be. It can become difficult for HCP’s to do their jobs efficiently as they may need to explain, interpret or suggest a conflicting opinion to the information presented from online resources [48]. Several HCP’s also believed that the internet poses risks as patients may misinterpret information and this can also cause unnecessary medical visits [28, 48].

*“For us, the doctors, the problem is that before starting you have to destroy. Patients come already with their theory and you have to dismantle it. It takes some care, and then you need to start anew.”* [48]*.*

### HCP’s reactions and behaviours to internet-informed patients

Patients/public found adopted reactions from HCP’s such as open body language and asking open questions, making the environment more comfortable and allowing them to feel more listened to, encouraging [28]. Many HCP’s agreed that it was important to show support to patients who used the internet for health information, but that such behaviours can bring associated tensions [38]. HCP’s agreed on the importance of collaboration with and guidance for patients, though they struggled to find the time to do this efficiently [38].

*“She smiled at me, she sort of sat there kind of just listening to everything, everything about her body language was just, you know, she was leaning forward, everything about it was just like really encouraging, really like, I’m here for you, I understand, I do recognise it, but don’t worry, don’t worry and she was able to tell me about her experiences as well.”* [27]*.*

HCP’s do sometimes experience anxiety around internet-informed patients, and some may find some of the information patients bring to consultations, slightly outside their area of expertise [38].

“*… because I think sometimes there’s a fear that patients expect you to know everything and sometimes it’s hard to admit that you don’t know.”* [28]*.*

### Sharing online health information with healthcare professionals

There were two key subthemes here: 1) Communication, which was seen as important in maintaining good relationships between patients and HCP’s. 2) Bringing online health information to the consultation, which was the decision making of whether patients would share their findings with their HCP. Six qualitative studies [27, 33, 38, 39, 45, 47], five quantitative studies [29, 32, 40, 41], and one mixed methods study [36], reported on this theme. This theme maps on to the collective action and reflexive monitoring constructs of NPT.

### Communication

Many studies explained that enabling better communication with HCP’s was one of several reasons why patients used the internet to explore health information [27, 38, 40, 45]. Townsend et al. [38] suggested that participants felt they gained more respect from HCP’s after seeking health information online as they were better prepared for their consultation and could make the most of the limited time. It also allowed them to communicate and interact better based on their increased background knowledge of the health conditions involved [27, 45].

*“You’re trying to communicate something to this person and you want the communication to be as effective as possible, so if you can show, if you can demonstrate that you understand something then that’s going to move the whole process.”* [27]

The HCP’s in Townsend et al. (2015) study agreed and said that it also allowed the consultation to be more interactive and direct as relevant questions could be asked. HCP’s also felt that patient preparation promoted more focused, effective, and efficient consultations [38].

### Bringing online health information to the consultation

There were several factors affecting whether patients chose to disclose or not disclose their access of internet health information to their HCP. Imes et al. (2008), found that some patients did not talk to their HCP about online health information as they did not trust the sources online. Others found that there was not enough time to bring up their research during consultations [32, 45]. Several patients did not want to tread on the HCP’s toes; such patients perceived they would be challenging the professional and did not want to question them or make them feel offended or intimidated by attempts to discuss online health information, thus interrupting the diagnostic process [27, 32, 47]. Other reasons for patients not discussing online health information research included feeling embarrassed and not wanting to be seen negatively [27, 32, 39]; such patients were concerned about HCP’s reactions to their health research online and felt that they might not be listened to or that the professionals might become dismissive or uninterested [27, 32, 36, 39]. In particular, it was found patients felt that physicians would not want patients to show them how to do their jobs [39]. In the survey by Russ et al. [41], 81% of respondents never showed their internet information to their doctors, although 77.9% were interested in their HCP referring them to appropriate online health websites. Rupert et al. [33] reported that some HCP’s discouraged future online health searches by indicating that the internet was an unreliable source.

*“As soon as I said I looked it up on the internet, he sort of leaned back, and sort of, [sigh] his shoulder dropped, and he, I didn’t feel that he was paying as much attention to me any more.”* [27]

In contrast to these perceptions of negative reactions, some patients felt that sharing health information they found online with their HCP’s could show that they had invested time and energy into the consultation; these respondents hoped this would promote them and their problems being taken more seriously [27, 45]. Positive experiences of patients sharing online health information with their doctors include all occasions when the doctor listens, acknowledges, and offers further discussion about such information [33]. Bartlett and Coulson [29] found most participants (82.2%) to be satisfied or extremely satisfied with their HCP’s reactions to their participation in online support groups, while a much smaller proportion experienced negative reactions (16.2%). They found that doctors’ body language was extremely important and that even a simple smile could change the dynamic of the conversation. Patients also hoped for acknowledgement of their efforts to participate in self-care [39]. Patients also brought up internet health information where they felt their research contradicted the physician’s interpretation [47]. However, many patients did not use the internet to replace HCP’s but rather to gain a deeper understanding of their symptoms or disease and to become more familiar with the appropriate terminology [36].

*“Because the fact that I actually go and research things on the internet, indicates to my GP that I’m actually serious about my health and I have an interest in it myself and I’m willing to take a bit more responsibility rather than just going in like a child, listening and being told what to do. I think it means that she’s more willing to treat me as an adult.”* [27]

### Impact of online medical searches and diagnosis on patient-healthcare professional relationships

There were three subthemes: trust; role changing and the patient-HCP relationship. Several studies have reported on the effects of this. Eight qualitative studies [27, 28, 30, 31, 46, 48, 50, 51], seven quantitative studies [32, 35, 42–44], and one mixed methods study [36], reported on this theme.

### Trust

Patients felt more trust in HCP’s and hoped for discussion regarding internet health information while seeking doctors’ opinions [27]. Patients felt more trusting towards their GP’s when they were honest about their levels of knowledge, acknowledging that generalists may not know some of the highly specific information provided online [27, 32]. Some HCP’s deliberately showed respect when presented with online health information as a way of making sure patients felt listened to and respected, in the hope of encouraging patients to continue self-care [28]. Adopting this approach allows more trust to develop between the patients and HCP’s [28]. One survey found that 57.5% of participants gave their physicians a perfect trust score but still used the internet after their visits to do further research [35]. Overall, health professionals were found to be more trustworthy and reliable than the internet [46].

*“I think that certain things should be left to doctors. That’s what they are there for! Even if the Internet helps us and gives us answers, the advice from my doctor gives me more confidence ( …*) *I trust my doctor 100%”* [50]*.*

HCP’s thus appear to be the most valuable source of health information [50]. Most studies emphasised that, regardless of the popularity of online self-diagnosis, the majority of respondents valued HCP’s opinions more, understood their explanations of diagnoses better, and had more trust in them [30, 50]. However, Hay et al. (2008), reported that 20% of participants went online to self-diagnose as they did not trust the diagnoses or treatment advice offered by their HCP’s [36].

### Role changing

Physicians have experienced changes in their roles since online health information has been introduced into consultations. Their new role can be described as acting as a partner to the patient, who is now more involved in both medical decision-making and consultation [47].

*“That’s what I’ve been experiencing by now for the last 20 years; my professional authority isn’t as sacred as it used to be. I can’t say anymore that’s it, that’s what I see, this is what we know and the patients are trusting and believe that we know best. It’s no longer like this.”* [47]*.*

*“You just have to be really open to the fact that they’re [patients] going to tell you things you didn’t know and that’s great. “Oh I hadn’t seen that before. That might be useful for me with other clients”. So I definitely feel it’s more of a partnership … [like] P2 says it’s much less didactic … Like P5 said, you just put in context what they’ve already brought to the table.”* [38]*.*

### The patient-HCP relationship

Some studies showed that HCP’s perceive internet health information as damaging to the patient-HCP relationship [48], though other studies found that most were satisfied with internet-informed patients [43]. It was found that nurses with higher self-epistemic authority and confidence, were less influenced by online health information presented to them than those with lower self-epistemic authority [42]. Barnoy et al. [44] also noted that nurses with higher computer self-efficacy and lower computer apprehensiveness had more positive attitudes towards internet-informed patients.

Many participants felt that online medical searching and self-diagnosis might cause misunderstandings between them and their HCP. They did not feel they were doing this to challenge the doctors’ credibility or capability in terms of diagnosis, and most patients prioritised the HCP’s advice over the information from the internet. However, where the HCP’s response to health information seeking is negative and disrespectful, this can seriously impact the patient-HCP relationship, and in some cases, this can lead to a patient changing their doctor or practice [27].

The results showed that most patients described their preferred role for HCP’s as being open-minded about online health communities and online health information seeking. They expected feedback on the validity of their research and recommendations for online health communities, allowing for more engagement in the decision-making process by the patient in conjunction with the HCP.

*“I was shocked out of my shoes the first time I went to the doctor, and the doctor came in and said, ‘Hi, my name is Steve. I’ll be your doctor, and I just want you to know that you are responsible for your health and I will make suggestions, and I would hope that you will take my suggestions, but it’s up to you. Your health is your concern.’ Wow! I mean it changes everything.”* [34]*.*

Patients tended to present information to the HCP to support the therapeutic relationship rather than to challenge it and Stevenson et al. [31] suggested that, based on this, HCP’s should check all such information for validity.

*“It’s just helped me have … more of a conversation with my doctor rather than just being, you know, have a one-sided, just listening. I feel like I can be more active in that interaction.”* [33].

Overall, the most common finding was that patients still prefer to see a HCP rather than performing online self-diagnosis and seeking internet health information. The internet is not seen as a replacement for visiting a HCP, but as offering a complimentary source of information [30, 50, 51].

## Discussion

The findings of our review demonstrate that although online self-diagnosis is a growing phenomenon, the public still tend to trust the advice from a HCP over any other information source and trust in HCP’s remained high. Nevertheless, the internet is viewed by patients as advantageous because of cost, accessibility, and the speed with which information can be obtained. Online resources were also viewed as valuable sources of emotional support and helpful resources to inform self-management and self-diagnosis of symptoms or conditions. It was also clear that most people did not feel that online self-diagnosis and health research had an impact on their relationship with HCP’s [27, 30, 31, 50, 51].

A large proportion of people found health information obtained online to be a complementary information source, that was an adjunct rather than substitute for HCP advice and treatment [30, 36, 47, 49, 50]. These people found the method of online self-diagnosis could be reassuring while waiting for a healthcare appointment that might be some time away [30]. People used information from the internet to become better informed about their health, to prepare for their consultation, to enable them to ask better questions and to help them better understand the information given to them by HCP’s [30, 38] thus helping to make consultations more productive for them.

It was clear from the findings that patients felt they had a better relationship with their HCP when they were able to discuss their online research with them and when their HCP responded positively to this. While, if people perceived a negative reaction from the HCP, this could cause distrust and embarrassment. HCP’s felt that if they disagree with information that the patient highly values, this may adversely affect the patient-HCP relationship [31, 47].

HCP’s reactions to people who had obtained health-related information from the internet were mixed; however, they were mostly positive. Some physicians felt that it was good that patients were looking after their own health, whereas others felt they were being challenged with information found online and that their patient had lost trust in them when they turned to the internet for help. Some also thought it could cause anxiety among patients – especially when the information was misinterpreted, and this could lead to unnecessary medical visits [28, 48].

Our findings also demonstrate that allowing patients to communicate health information obtained online with their HCP in the consultation, as well as the HCP showing that they value the patients’ research, can positively affect the relationship between the two. When HCP’s create an atmosphere that is open, this can encourage patients to discuss the information they have discovered; additionally the patient’s perception of invading the role of the HCP or their embarrassment may be reduced, which, overall, can enhance the relationship between the patient and HCP [28, 38].

There remain outstanding research gaps. The studies included in this mixed method systematic review mainly focused on the patient’s perspective of patients’ use of the internet for health-related research, making our results more focused on the patient’s evaluation. Fewer studies explored this from the HCP’s perspective. Very few studies (2/25) included the nurse’s perceptions of patients’ online self-diagnosis and online health research [42, 44]. with most focusing on doctors. The majority of studies focus on the stage of researching health information, but rarely considered perceptions of or reactions to self-diagnosis and the effects of this. Many studies were generalised for all health conditions, instead of focusing on just one or two health conditions. One study in this review focused solely on multiple sclerosis patients [36]. Few studies discussed the impact of online health forums and the effects they have on an individual and their perceptions, with only two of the studies included focusing on these groups [29, 33]. Future research should explore HCPs, particularly nurses’, perspectives on patients’ use of the internet for self-diagnosis and health research, particularly in the context of specific health conditions and on the effects of self-diagnosis.

Evidence from existing literature has previously suggested how online self-diagnosis can introduce a negative impact on the patient-HCP relationship [48] yet in contrast findings from this mixed methods systematic review has suggested that the internet can serve as a useful resource and can potentially improve the patient-HCP relationship if used in the correct way and high-quality sources are accessed.

### Strengths and limitations

Strengths of this review includes the systematic and rigorous approach taken to identify all relevant literature. A review protocol was published to PROSPERO [15] to enhance clarity and a robust thematic analysis with conceptualisation through a theoretical lens Normalisation Process Theory, to aid understanding. However, several limitations should be noted. Firstly, the search criterion that was used for this systematic review was broad in order to cover all areas that have been studied and that were associated with patient’s self-diagnosis and use of the internet. Unlike clinical type studies, where each condition or intervention has one universally used term, there is no consistently used terminology to describe the patient-HCP relationship and the aspects related to it. Only English language articles were searched for, which may have reduced the number of potentially relevant studies. Secondly, sources of information such as conference proceedings, theses and abstracts were not included, which means some related studies may have been missed.

## Conclusion

Although evidence has previously found that the internet can potentially have a negative impact on the patient-HCP relationship and can cause barriers in the relationship [48], this mixed methods systematic review has suggested, that patients’ use of the internet for self-diagnosis and health research has the potential to positively impact the HCP-patient relationship. Patients found HCP’s to be the most valued source of health information but found the internet to be a useful complementary tool [30, 36, 47, 49, 50]. Further research needs to be carried out in order to understand the effects that online health forums can have on the patient-HCP relationship so that all aspects of internet self-diagnosis can be thoroughly considered. There is also a need for more research on nurses and other AHPs perspectives of patients’ use of the internet for self-diagnosis and health-related research.

### Supplementary information

**Additional file 1.**

**Additional file 2.**

**Additional file 3.**

**Additional file 4.**

**Additional file 5.**

## Data Availability

The data that supports the results and findings of this systematic review can be found in either the main paper or the additional supporting files.

## Appendix

### Medline search syntax

1. exp Health Knowledge, Attitudes, Practice/
2. exp Internet/
3. exp Telemedicine
4. exp Medical Informatics
5. exp Information Systems/
6. exp Cell Phone/
7. (Tele?Health or digital health or eHealth).tw.
8. (computer assisted or cyber* or “e-health” or technolog* or (electronic adj health)).tw.
9. (mobilephone) or (mobile adj phone*) or cellphone or (cell adj phone*) or smartphone or (smart adj phone*)).tw.
10. (Mobile app* or twitter or facebook or social media or search engine* or online forum).tw.
11. (iPhone or i-phone or iphone or android).tw.
12. or/1-11
13. exp Information Seeking Behavior/
14. (Diagnos* or self?diagnos*).tw.
15. ((online or web* or internet) and diagnos* or self?diagnos*)).tw.
16. (Medical seeking or medical information or medical search or medical information website or symptom check*).tw.
17. (Health literacy or health information or health search or information seeking or health information website.tw.
18. or/13-17
19. exp Professional-Patient Relations/
20. (((Doctor* or nurs* or GP* or general practitioner* or physician* or healthcare professional* or health professional* or health-care professional* or healthcare provider*) and patient*) adj5 relation*).tw.
21. (physician* perception* or physician* opinion* or doctor* perception* or doctor* opinion* or nurs* perception* or nurs* opinion* or healthcare professional* perception* or healthcare professional* opinion* or health professional perception* or health professional opinion* or patient* perception* or patient* opinion*).tw.
22. or/19-21
23. 12 and 18 and 22
24. limit 23 to (english language and yr=“2007-Current”)

## Abbreviations

GPs: General Practitioners
HCP: Healthcare Professionals
NPT: Normalisation Process Theory
