# Supplementary material for: A mixed methods systematic review of the effects of patient online self-diagnosing in the ‘smart-phone society’ on the healthcare professional-patient relationship and medical authority
Source: BMC Med Inform Decis Mak. 2020 Oct 6;20:253. doi: 10.1186/s12911-020-01243-6 (PMC7539496; doi:10.1186/s12911-020-01243-6)
Supplement: Supplementary file 1 — Additional file 1. [file 12911_2020_1243_MOESM1_ESM.docx]

**Additional file 1: Qualitative data extraction table (Table 6)**

| **Author/Year/Country** | **Aims of study** | **Methods & Quality** | **Participants** | **Settings** | **Key findings** |
| --- | --- | --- | --- | --- | --- |
| **Ahluwalia et al. (2010), UK** | Examine the responses of GPs to internet prepared patients in consultations and what strategies GPs use for dealing with internet information being brought to the consultation. | Ethical consideration not reported. **Methodology:** Phenomenological. **Data collection method:** Semi-structured interviews. Framework approach to analysis used. MMAT = 3 (moderate). | Purposive sampling. 11 GPs. Five partners, three locum and three salaried doctors. Seven were white, three Asian and one Chinese. | Primary care GP practices. | GPs had a tendency to experience anxiety when patients brought health information from the internet to appointments but they were able to resolve the anxiety with several techniques. They learned to distance themselves from their emotional response and used cognitive and behavioural techniques to respond effectively to patients. Doctors perceived it as important to still feel of value to their patients as this was an effect on the doctor-patient relationship. |
| **Benetoli et al. (2018), Australia** | Explores the use of social media and its potential impact on patient’s interactions with healthcare professionals. | Ethical consideration not reported. **Methodology:** Phenomenology. **Data collection method:** 5 focus groups conducted. Thematic analytic approach used. MMAT = 4 (moderate-high). | 36 participants with a range of chronic conditions from hypertension, depression, arthritis and Crohn’s disease), on medications and use social media for health seeking purposes. | Setting not explicitly stated. | Participants did not tend to interact with healthcare professionals on social media and only used this to talk to peers. They reported they felt that the patient-HCP relationship had improved as they felt a sense of empowerment and increased knowledge from internet information. They mainly used it to prepare for consultations. |
| **Bowes et al. (2012), UK** | Explores patient’s reasons for presenting information at medical consultations and their perceptions of the GPs responses and what they wanted from their doctor. | **Qualitative**  Ethical consideration not reported. **Methodology:** Phenomenology. **Data collection method:** Semi-structured interviews. Inductive analytic approach. MMAT = 3 (moderate) | 26 participants. | Primary care – GP surgery. | Participants reported their use of the internet was to stay well informed about their health and make use of the time spent with the GP. Patients expected GPs to acknowledge their found information and discuss it. However, if the GP did not seem interested in their found information, patients reported damage to the doctor-patient relationship. Although, patients tended to value the opinion of the GP more than the internet. |
| **Caiata-Zufferey and Schulz. (2012), Switzerland** | Explores the strategies performed by physicians when interacting with internet informed patients and defines the motives behind their strategies. | Ethical approval obtained. **Methodology:** Phenomenology. **Data collection method:** Semi-structured interviews. Inductive analytic approach. MMAT = 5 (high). | 17 physicians. 5 general practitioners, 3 gynaecologists, 2 orthopaedic surgeons, 2 urologists, 2 oncologists, 1 allergist and 1 rheumatologist. | Primary care and medical specialist practices. | Results argue that physician’s communicative strategies are partly ‘personality dependent’ or ‘context-dependent’. Most physicians in this study found their consultations to be disrupted by patients who introduce online health information. It is reported that the internet can impact the patient-doctor relationship depending on the physician’s responses and the patient’s expectation. |
| **Chu et al. (2017), China** | Explores individual’s perceptions of online health information seeking and to understand their behaviours. | **Qualitative**  Ethical approval obtained. **Methodology:** Phenomenology.  **Data collection method**: Five focus groups. Thematic and in-depth analytic approaches used. MMAT = 4 (moderate). | 49 participants aged 18 years or above and residing in Hong Kong. | Community | Older adults (55+) were less likely to use the internet to find health information. The main reasons for using the internet was because of limited time with the doctors, barriers to accessing professional health services and it was convenient. Although, the participants reported they limited trust in some online websites and it caused some frustration and fear. However, regardless of the severity of the health issue, the internet was always the first source of information to be accessed. |
| **Donnelly et al. (2008), UK** | Explores the use of the internet and e-health amongst adults and their attitudes and reasons for using the internet for health information. | Ethical approval obtained. **Methodology:** Phenomenology. **Data collection method:** 4 focus groups with an open-ended schedule. Thematic analytic approach. MMAT = 4 (moderate). | 16 participants recruited through quota convenience sampling. | Setting not stated. | Three themes developed from the data: decline in expert authority, pervasiveness of health information on the internet and empowerment. Participants tended to like the immediate benefits of e-health and felt empowered by increasing their knowledge however, they would be reluctant to lose face-to-face consultations with their GP over it. |
| **Macias and McMillan. (2008), USA** | Investigates how older adults use the internet to seek health information and health communication. | Ethical consideration not reported. **Methodology:** **Data collection method:**  Focus groups. Corbin & Strauss (1990) method of analysis. MMAT = 3 (moderate). | 31 participants recruited through snowballing techniques. | Community Centres. | Participants found that information that is easily accessible is not reliable or of high quality. Participants also did not trust advertising websites. They prefer collecting information from government websites or educational institutions. |
| **Mendes et al. (2017), Portugal** | Explores the way young adults search for health information online and how they rank the sources of information by credibility and reliability. | Ethical consideration not reported. **Methodology:** Phenomenology. **Data collection method:**  15 qualitative interviews. Grounded theory approach undertaken. MMAT = 4 (moderate-high). | 15 participants recruited from recruited from administrative staff at their routine follow-up appointments. | Epidemiology department of a medical school of a public Portuguese University. | The findings had shown that the participants found themselves committed to online health information but found healthcare professionals more resourceful. Although they found health information on the internet useful, they also felt it could be seen as unreliable. |
| **Rupert et al. (2014), USA** | Explores individual’s use of information from online health communities in medical consultations and examining how healthcare professionals react to this. | Ethical approval obtained. **Methodology:** Phenomenology. **Data collection method:**  10 in-person and virtual focus groups. Thematic analytic approach. MMAT = 3 (moderate). | 89 Patients and caregivers who visited online health communities. | Online community. | Participants felt that online health communities provided more detailed information that the healthcare professional may not have had time to give. Patients and caregivers reported that they did not only want to learn about the medical aspects of their illness, but the emotional and logistical aspects as well. Although, it is suggested that online health communities can be used as a facilitator instead of a barrier for shared decision making between patients/caregivers and the healthcare professional. |
| **Silver (2015), Canada** | Explores patient’s problems when going online to treat a health issue and identifies barriers when communicating this information with doctors. | Ethical approval obtained. **Methodology:** Exploratory study. **Data collection method:** semi-structured interviews about opinions and experiences of online health information seeking. Inductive content and thematic analytic approach to qualitative data. MMAT = 5 (high). | 56 participants aged 50 years and over. Recruited through brochures that were advertised in eight randomly selected neighbourhoods. | Community | 36% of participants were concerned with non-physical harm that could happen from internet diagnosing and 29% had concerns with their anxiety. Participants felt sharig online health information with their doctors was embarrassing and not appropriate. Findings supported the need for doctors to try and guide the patients to high-quality online health information websites and in some cases, initiate the conversation about online health information seeking. |
| **Sommerhalder et al. (2009), Switzerland** | Analyses the benefits and difficulties of online health information from the patient and physician perspective. | Ethical approval requested but formal approval was not necessary for this study. **Methodology:** Grounded theory. **Data collection method:** Semi-structured interviews. MMAT = 5 (high). | Patients and physicians from primary care and medical specialist practices. 32 patients and 20 physicians. General practitioners (n=12) and specialists (n=8). | Primary care and medical specialist practices. 104 randomly selected medical practices to recruit physicians. | Findings showed that physicians were happy to discuss online health information with patients during consultations although not all patients discussed this in consultations to avoid any conflicts and lack of time. |
| **Stevenson et al. (2007), UK** | Explored patient’s views on the effects the internet has on the patient-doctor relationship. | Ethical approval obtained. **Methodology:**  **Data collection method:**  8 disease specific focus groups. Subsequent analytic approach. MMAT = 5 (high). | 34 adult patients. Adult patients with diabetes mellitus, hepatitis C or ischaemic heart disease. | Clinic and community settings. | The results showed that patients seeking health information online is not to disrupt the balance of power or roles in the consultation but instead used as an additional source for patients. Doctors should not feel challenged by patients bringing online health information to consultations and should instead see it as the patient trying to work alongside the doctor with hope for them to respond positively. |
| **Townsend et al. (2015), Canada** | Explores the influence of different types of eHealth use and how internet health information can impact the patient-HCP relationship. | Ethical considerations not reported. **Methodology:** Grounded theory. **Data collection method:** Focus group discussions recruited through online arthritis sites, web and social media sites such as Facebook and Twitter. An iterative, thematic analysis approach was undertaken. MMAT = 5 (high). | 18 patients with arthritis and co-conditions, along with 14 healthcare professionals (physical and occupational therapists, rheumatology nurse, laboratory technician, rheumatology fellows, physicians and rehabilitation providers | Community centres, health care centres or participants work. | Patients had a tendency to go online for information about diagnosis and to find others experiencing similar symptoms. Health related internet information can support patient decision making and empowerment however, can also cause tension in the relationship – such as time constraints or difference in opinion. Healthcare professionals have learned techniques to handle negotiation in the consultation and to avoid conflicts. |
| **Lee et al. (2014), Australia** | Explores the navigational needs of the public when searching for internet health information for self-management of chronic health conditions. | Ethical approval obtained. **Methodology:** **Data collection method:** semi-structured face to face interviews. Thematic analysis undertaken. MMAT = 4 (moderate-high). | Purposive sampling conducted. 17 participants in total. Participants were 18 years and over with at least one chronic health condition and used the internet to search for health information. | Community – nine public pharmacies. | Participants wanted to be able to navigate the internet better to search for health information. Online health information seeking was most commonly done after consultations with the health professional. Patients found online health information most commonly through search engines (Google). The study suggests more involvement by health professionals regarding online health information can benefit the patient. |
| **Caiata-Zufferey et al. (2010), Switzerland** | Explores the process of patient’s online health information searching before or after a medical consultation. | Ethical approval obtained. **Methodology:** Grounded theory. **Data collection method:** semi-structured interviews. Comparative analysis adopted. MMAT = 5 (moderate-high). | Purposive sampling conducted. 27 patients with a variety of health conditions. | 14 medical practices. | Findings reported that patients search online for health information to prepare for the medical consultation. Motivations as to why people were searching online were for acknowledgement, perspective and reduction of uncertainty. |
| **Fiksdal et al. (2014), USA** | Explores the perceptions of community members in relation to online health information seeking activities. | Ethical approval obtained. **Methodology: Data collection method:** three focus groups. Grounded theory approach to analysis. MMAT = 4 (moderate). | 19 participants who are all residents of Olmsted County, Minnesota and are either mayo clinic patients, employees or have at least one family member who is a patient or employee. | Setting not stated. | Most patients found the internet to be a valuable tool to find information and it helped with their preparation for consultations. Patients viewed online health information seeking as a way to build the patient-doctor relationship. |
