# Supplementary material for: A mixed methods systematic review of the effects of patient online self-diagnosing in the ‘smart-phone society’ on the healthcare professional-patient relationship and medical authority
Source: BMC Med Inform Decis Mak. 2020 Oct 6;20:253. doi: 10.1186/s12911-020-01243-6 (PMC7539496; doi:10.1186/s12911-020-01243-6)
Supplement: Supplementary file 2 — Additional file 2. [file 12911_2020_1243_MOESM2_ESM.docx]

**Additional file 2: Quantitative data extraction table (Table 7)**

| **Author/Year/Country** | **Aims of study** | **Methods & Quality** | **Participants** | **Settings** | **Key findings** |
| --- | --- | --- | --- | --- | --- |
| **Barnoy et al. (2008), Israel** | Examined nurse’s attitudes towards internet informed patients and elements that may influence their attitude. | Ethical approval obtained. **Methodology:** Survey. **Data collection method:** Questionnaire. Regression analysis conducted. MMAT = 2 (low). | 110 hospital nurses (32 practical nurses, 35 registered nurses and 43 with a BA nursing degree). Aged between 21-49 years. | Hospital nurses | Nurses with more experience had a better attitude towards online health information than those with less experience. The findings showed that the more computer literate nurses are, the more positive their attitudes are about patients presenting with internet health information. |
| **Barnoy et al. (2011), Israel** | Explored nurse’s reactions to online health information that was retrieved from sources of different credibility and the link between self-epistemic authority and the reactions. | Ethical approval obtained. **Methodology:** Survey. **Data collection method:** Questionnaire. T-tests were used to carry out analysis. MMAT = 2 (low). | (N=101) female hospital staff nurses | Tel Aviv University OR hospital | Nurses that had obtained an academic degree, had a more positive attitude to internet health information seeking as opposed to nurses who did not have an academic degree. Nurses reactions to different sources of internet information vary on their education and level of self-epistemic authority. |
| **Bartlett and Coulson. (2011), UK** | Explores the effects that online support groups may have on the patient-health care professional relationship and patient empowerment. | Ethical considerations not reported. **Methodology**: Survey **Data collection method:** Online questionnaire. Descriptive statistics and binary and multiple logistic regressions used for analysis. MMAT = 2 (low). | 246 participants recruited from 33 chronic illness online support groups. | Chronic illness online support groups. | 82.2% of participants had discussed information they found online with their health professional and 74.2% were satisfied with how the health professional responded. Although, 60.3% felt being a member of an online support group has affected their relationship with health professionals. |
| **Bell et al. (2011), USA** | Examines the reasons why patients seek health information online and the predictors and prevalence of online health information seeking after a medical appointment. | Ethical approval obtained. **Methodology:** Online survey. **Data collection method:** Questionnaire. Descriptive statistics and logistic regression analysis. MMAT = 2 (low). | Non-random convenience sample of 274 respondents. Recruited from an online community - ‘Daily Strength’. | Online health community. | 60.8% went online to search for information post medical visit. Going online was identified with issues with trust (P=.002) and increased worrying (P=.049). The most common reason for these searches were for being curious (71%) and also not being satisfied with the physician’s behaviour (32%). |
| **Giveon et al. (2009), Israel** | Evaluate the reactions of primary care physician’s reactions in response to internet informed patients and to assess the influence of physician’s personal and demographic characteristics on their satisfaction with e-patients. | **Quantitative**  Ethical considerations not reported. Cross-sectional design. **Methodology:** Survey. **Data collection method:** 17 item questionnaire. SPSS-PC software used and chi-square.  MMAT = 3 (moderate). | Convenience sample of 118 primary care physicians from rosters of Clalit Health Services. | Primary care clinics and medical education course at Tel Aviv University. | 66.7% of physicians were satisfied with the quality of online health information that their patients brought. 88.7% were content with their relationships with patients and with the knowledge they collected from their patients and their use of online information. Most physicians found it favourable for patients to bring information from the internet to consultations and demographic variables did not affect physician behaviour with e-patients. |
| **Haluza et al. (2017), Austria** | Explores the public perceptions of online health information seeking and the impact this has on the doctor-patient relationship. | Ethical approval obtained.  Cross sectional study **Methodology:** Online survey. **Data collection method:** Questionnaire. Descriptive statistics to report quantitative data. MMAT = 2 (moderate). | Convenience sample of 562 participants. | Setting not stated. | Digital immigrants (38.9%) found data exchange to be more acceptable than digital natives (30.8%). 55.2% reported visiting online health websites after a doctor’s consultation – digital natives more likely (59.7% vs. 49.8%). Overall, the internet was the most used health information source amongst digital generations although, doctors still remained the most preferred source of health information. |
| **Imes et al. (2008), USA** | Explores the possibilities of why patients may resist discussing online health information with their healthcare providers. | Ethical approval obtained. **Methodology:** Online survey over a 6-month period. **Data collection method:** 11 closed-ended items, 4 open-ended itemed and demographic information questionnaires. Reliability measured using Cohen’s kappa. MMAT= 3 (moderate). | 714 participants recruited from internet health message boards. | Online setting. | 20.2% refrained from sharing online health information with their healthcare provider as they reported they were searching for their own personal benefit and a further 10% didn’t share as they did not trust the resource. 13% did not want to step on the provider’s ‘turf’ therefore, did not disclose their found information. 8.2% felt embarrassed to show it and 14.4% felt they would be dismissed or that the healthcare provider would be uninterested in their findings. |
| **Russ et al. (2011), Israel** | Examined internet use for health information seeking by primary care patients and their perceptions of effects internet use may have on their relationship with the doctor. | Ethical considerations not reported.  **Methodology:** Cross-sectional survey. **Data collection method:** Questionnaire. Analysed using EPI-INFO software and tested using chi-square and t-tests. MMAT= 4 (moderate-high). | Convenience sample of 138 patients visiting primary care clinics. | 10 primary care clinics. | 89% had internet access and 41% used the internet as a source for health information. Although, most patients did not share the information they found with their doctor (81%). Although, those who did believed it had a positive impact on their relationship with the doctor (87%). 77.9% of patients would be interested in doctors referring them to quality health information websites. |
