# Supplementary material for: A mixed methods systematic review of the effects of patient online self-diagnosing in the ‘smart-phone society’ on the healthcare professional-patient relationship and medical authority
Source: BMC Med Inform Decis Mak. 2020 Oct 6;20:253. doi: 10.1186/s12911-020-01243-6 (PMC7539496; doi:10.1186/s12911-020-01243-6)
Supplement: Supplementary file 3 — Additional file 3. [file 12911_2020_1243_MOESM3_ESM.docx]

**Additional file 3: Mixed methods data extraction table (Table 8)**

| **Author/Year/Country** | **Aims of study** | **Methods & Quality** | **Participants** | **Settings** | **Key findings** |
| --- | --- | --- | --- | --- | --- |
| **Hay et al. (2008), USA** | Explores the online information seeking of multiple sclerosis (MS) patients and their reasons for doing so, and the importance of physician-patient communication about this information. | Ethical approval obtained. Semi-structured interviews, before and after appointments for patients presenting at the clinic for the first time. 3-item post appointment questionnaire on physician’s satisfaction with appointment.  MMAT = 3 (moderate). | (N=61) participants. 79% female and 21% male. | MS clinic. | 82% of participants collected online health information before their first appointment and 36% discussed the information with their physician. A reason for not showing the physician the information was because of wariness of health care and potentially leading to non-adherence. |
