# Supplementary material for: A mixed methods systematic review of the effects of patient online self-diagnosing in the ‘smart-phone society’ on the healthcare professional-patient relationship and medical authority
Source: BMC Med Inform Decis Mak. 2020 Oct 6;20:253. doi: 10.1186/s12911-020-01243-6 (PMC7539496; doi:10.1186/s12911-020-01243-6)
Supplement: Supplementary file 4 — Additional file 4. [file 12911_2020_1243_MOESM4_ESM.docx]

**Additional file 4: Sociodemographic table (Table 9)**

| **Author/Year/Country** | **No. of participants** | **Types of participants** | **Gender** | **Age** | **Ethnicity** | **Socioeconomic status** |
| --- | --- | --- | --- | --- | --- | --- |
| **Townsend et al (2015), Canada** | 18 patients and 14 healthcare professionals. | Patients with arthritis and at least one other condition. HCPs included physical and occupational therapists, rheumatology nurse, laboratory technician, rheumatology fellows and physicians and rehabilitation providers. | Patients: Female (n=16) Male (n=2) Healthcare professionals: Female (n=11) Male (n=3) | Patients: between 30-70 years old.  Healthcare professionals: between 30-60 years old. | Majority Caucasian. | Majority was middle-class and Caucasian. |
| **Haluza et al (2017), Austria** | 562 | Public | Female (n=331) Male (n=231) | Mean age was 36.9. | Vienna | Education: Primary (n=90) Secondary (n=191) Tertiary (n=281)  Healthcare professional (n=243), Non-healthcare professional (n=319). |
| **Imes et al (2008), USA** | 714 | Participants recruited from internet health message boards. | Female= 89.3% Male= 10.7% | Mean age = 43 years. | Majority of participants lived in Vienna. | Median range for household income = $40,000-59,000.  Lived in urban areas = 58%, suburban areas = 18%, small towns = 16%, rural areas = 8% |
| **Bowes et al (2012), UK** | 26 | Participants who reported online health information to their GP. | Female (n=16) Male (n=10) | Participants all over 18 years old. | White (n=19) Other (n=7) | Education: Degree level or higher (n=18). |
| **Russ et al (2011), Israel** | 138 | Patients visiting the selected 10 primary care clinics. | Female (n=82) Male (n=53) | Mean age = 38.7 years. | Israel | Education: Elementary (n=5) High school (n=34) University (n=96) Income: < national average (n=21) Average (n=78) > average (n=31) |
| **Benetoli et al (2018), Australia** | 36 | Patients with chronic conditions and on medications who have used social media for health reasons. | Female (n=17) Male (n=19) | Age range= 27-71 years Mean age= 47.3 years. | Birth country: Australia (n=26) England (n=2) New Zealand (n=2) Other (n=6) | Education: < high school (n=3), high school (n=11), college or technical education (n=6), undergraduate (n=12), postgraduate (n=2), missing data (n=2). Employment: Full time (n=18), part time (n=9), home duties (n=3), retired (n=2), unemployed (n=4). |
| **Sommerhalder et al (2009), Switzerland** | Patients (n=32) Physicians (n=20) Total: (n=52) | Patients and physicians from primary care and medical specialist practices. General practitioners (n=12) and specialists (n=8). | Patients: Female (n=12) Male (n=20) Physicians: Female (n=4) Male (n=16) Female patients= 12 | Age range from 19-79. Mean age= 49 | Ethnicity not described. | < 12 years of education (n=17) High school (n=13). |
| **Caiata-Zufferey & Schulz (2012), Switzerland** | 17 physicians | Physicians from primary care and medical specialist practices. General practitioners (n=5), gynaecologists (n=3), orthopaedic surgeons (n=2), urologists (n=2), oncologists (n=2), allergist (n=1), endocrinologist (n=1), rheumatologist (n=1). | Female (n=3) Male (n=14) | Aged between 40 and 64 years. Mean age = 52. | Ethnicity not described. | Socioeconomic status not reported. |
| **Barnoy et al (2011), Israel** | 101 female hospital staff nurses. | Registered nurses with bachelor degree in nursing (n=36). Registered nurses without academic degree (n=65). | Female (n=101) Male (n= 0) | Mean age = 30.9 | Birth place: Israel (n=17), Former Soviet Union (n=29), Asia (n=4), Europe (n=3) | Socioeconomic status not reported. |
| **Ahluwalia et al (2010), UK** | 11 general practitioners | General practitioners: five partners, three locums and three salaried doctors. | Female (n=6) Male = (n=5) | Age not reported. | White (n=7) Asian (n=3) Chinese (n=1) | Socioeconomic status not reported. |
| **Giveon et al (2009), Israel** | 118 physicians | Primary care physicians, board certified specialists in family medicine, general practitioners without board certification and final year family medicine residents working in own practice. | Female= (n=39) Male= (n=79) | Mean age = 49 years | Place of birth: Israel (n=71) Eastern Europe (n=33) Other (n=14) | Socioeconomic status not reported. |
| **Barnoy et al (2008), Israel** | 110 hospital nurses | Practical nurses (n=32), registered nurses (n=35), academically trained nurses (n=43). | Female (n=101) Male (n= 9). | Age range from 21-49 years. Mean age = 30.83. | Immigrants from the former Soviet Union = 60%, native born Israelis = 33%, other countries = 7%. | Socioeconomic status not reported. |
| **Rupert et al (2014), USA** | 89 | Patients/caregivers who use online health community groups. | Female (n=58) Male (n=31) | 18-24 (n= 6) 25-34 (n= 16) 35-44 (n= 20) 45-54 (n= 27) 55-64 (n=16) 65-74 (n= 4) | Caucasian (n=62), African American (n=17), Hispanic (n=2), Asian (n=2), Hawaiian or Pacific Islander (n=2), Other (n=4). | < High school (n=1), high school (n=3), some college or technical school (n=22), college graduate (n=39), some graduate school (n=6), graduate school degree (n=17). |
| **Bartlett & Coulson (2011), UK** | 246 | Chronic illness online support groups. | Females (n= 174) Males (n=72) | Age range from 21-100, Mean age = 50.41 | Ethnicity not described. | Socioeconomic status not reported. |
| **Silver (2015), Canada** | 56 | Participants over 50 years old, have regular contact with primary care physician or general practitioner and use the internet regularly for health-related issues. | Female (n=30) Male (n=26) | Mean age = 69 | Born in Canada (n=32) Other (n= 24) | Education: <High school (n=7), completed high school (n=11), college/university (n=14), graduate school (n=24).  Income: Income > Canadian $60,000 (n=26). |
| **Donnelly et al. (2008), UK** | 16 | Not described. | Female (n=8) Male (n=8) | Age range from 19-62 years. Mean age = 37.5 years. | All participants white British. | Socioeconomic status not reported. |
| **Mendes et al. (2017), Portugal** | 15 | Health individuals born in 1990 with no diagnosis or at risk of a medical condition. | Female (n=8) Male (n=7) | Age not reported. | All participants were white European. | All lived in metropolitan area of northern Portuguese city. All university students except one who completed 6 years of high school. |
| **Macias and McMillan. (2008), USA** | 31 | Participants age 60 or over and use the internet for more than just emails. | Female (n=15) Male (n=16) | Age range from 63-83 years. Mean age = 72.80. | Ethnicity not described. | Socioeconomic status not reported. |
| **Chu et al. (2017), China** | 49 | Not described. | Female (n=23) Male (n=26) | 18-24 (n=9) 25-34 (n=8) 35-44 (n=8) 45-54 (n=6) 55-64 (n=8) 65+ (n=8) | Ethnicity not described. | Education: Primary or below (n=2), secondary (n=14), tertiary or above (n=31).  Monthly income: <10,000 (n=7), 10,000-19,999 (n=7), 20,000-29,999 (n=8), 30,000-39,999 (n=4), 40,000+ (n=12). |
| **Bell et al. (2011), USA** | 274 | Internet support community members. | Female (n=227) Male (n=47) | 18-29 (n=45) 30-39 (n=49) 40-49 (n=64) 50-59 (n=77) 60-69 (n=35) ≥70 (n=4) | White (n=256) Other (n=18) | Education: High school graduate/or less (n=45), A.A./tech degree or some college (n=127), college graduate (n=102).  Income: Low- ≤$40,000 (n=110), Moderate - >$40,000 to ≤$80,000 (n=92), High >$80,000 (n=62), refused to answer (n=10). |
| **Hay et al. (2008), USA** | 61 | Patients attending an MS clinic for the first time. | Female (n=49) Male (n=12) | 0-30 (n=11) 30-50 (n=33) >50 (n=16) | Ethnicity not described. | Education: ≤$40,000 (n=9), $40,000-$100,000 (n=29), >$100,000 (n=21). |
| **Stevenson et al. (2007), UK** | 34 | Patients with diabetes mellitus, ischaemic heart disease or hepatitis C. | Female (n=12) Male (n=22) | 30-39 (n=3) 40-49 (n=4) 50-59 (n=7) 60-69 (n=16) 70-79 (n=4) | White British (n=26), White European (non-British origin) (n=5), Asian or British Asian (n=2), Black or Black British (n=1). | Education: School leaver (n=8), A levels or equivalent (n=7), Degree, HND or similar (n=17), not disclosed (n=2). Employed (n=8), economically active (n=26). |
| **Lee et al. (2014), Australia** | 17 | Patients with chronic health conditions. | Female (n=9) Male (n=8) | Age ranges from 19-85 years. Most common age category between 50-60 years. | Ethnicity not described. | University students (n=3) Workforce (n=8) Retirees (n=6). |
| **Caiata-Zufferey et al. (2010), Switzerland** | 27 | Patients that had search for health information online that related to a problem they discussed in the medical encounter. | Female (n=16) Male (n=11) | Age ranges from 21-69 years. Mean age was 43 years. | Ethnicity not described. | Education:  Completed secondary school (n=2) Certificate of apprenticeship (n=12) Completed high school or equivalent (n=5) University degree (n=8)  Work:  Paid job (n=18) Homemakers (n=7) Retired (n=1) Unemployed (n=1) |
| **Fiksdal et al. (2014), USA** | 19 | Mayo clinic patients, employees and family visitors. | Female (n=14) Male (n=5) | Mean age = 43.26 years | White (n=15) Black or African American (n=0) Asian (n=4) | Education: High school or GED (n=0), community or junior college (n=3), four-year college (n=3), graduate school (n=13).  Household income: $15,000-$35,000 (n=2), $35,001-$55,000 (n=9), $55,001-$75,000 (n=4), $75,001-$100,000 (n=0), over $100,000 (n=1), prefer not to say (n=3). |
